# Inpatient antibacterial use trends and patterns, China, 2013–2021

**DOI:** 10.2471/BLT.22.288862

**Published:** 2023-01-18

**Authors:** Haishaerjiang Wushouer, Yue Zhou, Wanmeng Zhang, Lin Hu, Kexin Du, Yaoyao Yang, Guiqing Yao, Paul Little, Bo Zheng, Xiaodong Guan, Luwen Shi

**Affiliations:** a Department of Pharmacy Administration and Clinical Pharmacy, School of Pharmaceutical Sciences, Peking University, 38 Xueyuan Road, Haidian District, Beijing, 100191, China.; b Department of Health Sciences, University of Leicester, Leicester, England.; c Primary Care Population Sciences and Medical Education Unit, University of Southampton, Southampton, England.; d Institute of Clinical Pharmacology, Peking University First Hospital, Beijing, China.

## Abstract

**Objective:**

To analyse trends and patterns in inpatient antibacterial use in China’s tertiary and secondary hospitals between 2013 and 2021.

**Methods:**

The analysis involved quarterly data from hospitals covered by China’s Center for Antibacterial Surveillance. We obtained information on hospital characteristics (e.g. province, a de-identified hospital code, hospital level and inpatient days) and antibacterial characteristics (e.g. generic name, drug classification, dosage, administration route and usage volume). We quantified antibacterial use as the number of daily defined doses per 100 patient-days. The analysis took into account the World Health Organization’s (WHO’s) Access, Watch, Reserve classification of antibiotics.

**Findings:**

Between 2013 and 2021, overall antibacterial use in inpatients decreased significantly from 48.8 to 38.0 daily defined doses per 100 patient-days (*P* < 0.001). In 2021, the variation between provinces was almost twofold: 29.1 daily defined doses per 100 patient-days in Qinghai versus 55.3 in Tibet. The most-used antibacterials in both tertiary and secondary hospitals throughout the study period were third-generation cephalosporins, which comprised around one third of total antibacterial use. Carbapenems entered the list of most-used antibacterial classifications in 2015. The most frequently used antibacterials in WHO’s classification belonged to the Watch group: usage increased significantly from 61.3% (29.9/48.8) in 2013 to 64.1% (24.4/38.0) in 2021 (*P* < 0.001).

**Conclusion:**

Antibacterial use in inpatients decreased significantly during the study period. However, the rising proportion of last-resort antibacterials used is concerning, as is the large gap between the proportion of antibacterials used belonging to the Access group and WHO’s global target of no less than 60%.

## Résumé

**Objectif:**

Analyser les tendances et schémas propres à l'utilisation des antibactériens en milieu hospitalier dans les hôpitaux secondaires et tertiaires en Chine, entre 2013 et 2021.

**Méthodes:**

L'analyse repose sur des données trimestrielles provenant d'hôpitaux couverts par le Center for Antibacterial Surveillance chinois. Nous nous sommes procuré des informations sur les caractéristiques des hôpitaux (province, code hospitalier anonymisé, niveau de l'établissement et jours d'hospitalisation par exemple) et des antibactériens (notamment le nom générique, la classe pharmacologique, le dosage, le mode d'administration et le volume utilisé). Nous avons quantifié l'usage d'antibactériens et le nombre de doses journalières définies par 100 jours-patients. L'analyse a pris en compte la classification AWaRe (Access, Watch et Reserve) de l'Organisation mondiale de la Santé.

**Résultats:**

Entre 2013 et 2021, le recours global aux antibactériens en milieu hospitalier a considérablement diminué, passant de 48,8 à 38,0 doses journalières définies pour 100 jours-patients (*P* < 0,001). En 2021, les variations entre provinces étaient presque multipliées par deux: 29,1 doses journalières définies pour 100 jours-patients dans le Qinghai, contre 55,3 au Tibet. Pendant toute la durée de l'étude, les antibactériens d'usage le plus courant, tant dans les hôpitaux secondaires que tertiaires, étaient les céphalosporines de troisième génération, qui représentaient près d'un tiers de l'usage total d'antibactériens. Les carbapénèmes ont été ajoutés à la liste des classes d'antibactériens les plus utilisées en 2015. D'après la classification de l'OMS, les antibactériens les plus fréquemment employés appartenaient à la catégorie Watch: leur usage a sensiblement augmenté, passant de 61,3% (29,9/48,8) en 2013 à 64,1% (24,4/38,0) en 2021 (*P* < 0,001).

**Conclusion:**

L'emploi d'antibactériens en milieu hospitalier a connu une forte baisse au cours de la période d'étude. Cependant, le pourcentage d'utilisation croissant des antibactériens de dernier recours est inquiétant, tout comme l'important décalage entre le pourcentage d'antibactériens utilisés appartenant à la catégorie Access et la cible mondiale de l'OMS, qui consiste à ne pas dépasser les 60%.

## Resumen

**Objetivo:**

Analizar las tendencias y patrones en el uso de antibacterianos en pacientes hospitalizados en hospitales de atención terciaria y secundaria de China entre 2013 y 2021.

**Métodos:**

El análisis incluyó datos trimestrales de hospitales cubiertos a través del Center for Antibacterial Surveillance (Centro de Vigilancia Antibacteriana) de China. Se obtuvo información sobre las características de los hospitales (por ejemplo, la provincia, un código de hospital anónimo, el nivel del hospital y los días de hospitalización) y las características de los antibacterianos (por ejemplo, el nombre genérico, la clasificación del fármaco, la dosis, la vía de administración y el volumen de uso). Se cuantificó el uso de antibacterianos como el número de dosis diarias definidas por cada 100 pacientes-día. El análisis tuvo en cuenta la clasificación de antibióticos Access (Acceso), Watch (Vigilancia) y Reserve (Reserva) de la Organización Mundial de la Salud (OMS).

**Resultados:**

Entre 2013 y 2021, el uso general de antibacterianos en pacientes hospitalizados disminuyó de manera significativa de 48,8 a 38,0 dosis diarias definidas por cada 100 pacientes-día (*p* < 0,001). En 2021, la variación entre provincias fue de casi el doble: 29,1 dosis diarias definidas por cada 100 pacientes-día en Qinghai frente a 55,3 en Tíbet. Los antibacterianos más utilizados tanto en hospitales de atención terciaria como secundaria durante todo el periodo de estudio fueron las cefalosporinas de tercera generación, que representaron alrededor de un tercio del uso total de antibacterianos. Los carbapenémicos se incorporaron a la lista de antibacterianos más utilizados en 2015. Los antibacterianos más utilizados en la clasificación de la OMS pertenecían al grupo Watch: el uso aumentó a un ritmo significativo del 61,3 % (29,9/48,8) en 2013 al 64,1 % (24,4/38,0) en 2021 (*p* < 0,001).

**Conclusión:**

El uso de antibacterianos en pacientes hospitalizados disminuyó en gran medida durante el periodo de estudio. Sin embargo, el aumento del porcentaje de antibacterianos de último recurso utilizado es preocupante, al igual que la gran diferencia entre el porcentaje de antibacterianos utilizados pertenecientes al grupo Access y el objetivo global de la OMS de no menos del 60 %.

## ملخص

**الغرض:**

تحليل الاتجاهات والأنماط في استخدام مضادات البكتيريا للمرضى الداخليين في المستشفيات الثالثة والثانوية في الصين بين عامي 2013 و2021.

**الطريقة:**

شمل التحليل البيانات ربع السنوية من المستشفيات التي يغطيها المركز الصيني لمراقبة مضادات البكتيريا. حصلنا على معلومات عن خصائص المستشفيات (مثل المقاطعة، ورمز المستشفى غير المحدد لهويتها، ومستوى المستشفى، وأيام المرضى الداخليين)، والخصائص المضادة للبكتيريا (مثل الاسم العام، وتصنيف دواء، والجرعة، ومسار الإدارة، وحجم الاستخدام). قمنا بتقدير كمية استخدام مضادات البكتيريا كعدد الجرعات اليومية المحددة لكل 100 مريض/يوم. أخذ التحليل في الاعتبار تصنيف منظمة الصحة العالمية (WHO) للوصول، والمراقبة، والاحتياطي للمضادات الحيوية.

**النتائج:**

بين عامي 2013 و2021، انخفض الاستخدام الكلي لمضادات البكتيريا في المرضى الداخليين بشكل ملحوظ من 48.8 إلى 38.0 جرعة محددة يوميًا لكل 100 مريض/يوم (نسبة الاحتمال < 0.001). في عام 2021، كان الاختلاف بين المقاطعات مزدوجًا تقريبًا: 29.1 جرعة محددة يوميًا لكل 100 مريض/اليوم في شنغهاي مقابل 55.3 في التبت. كانت مضادات البكتيريا الأكثر استخدامًا في كل من المستشفيات الثالثة والثانوية خلال فترة الدراسة هي الجيل الثالث من السيفالوسبورينات، والتي شكلت حوالي ثلث إجمالي استخدام مضادات البكتيريا. دخلت الكاربابينيمات قائمة التصنيفات المضادة للبكتيريا الأكثر استخدامًا في عام 2015. تنتمي مضادات الجراثيم الأكثر استخدامًا في تصنيف منظمة الصحة العالمية إلى مجموعة المراقبة: زاد الاستخدام بشكل كبير من %61.3 (29.9/48.8) في عام 2013 إلى %64.1 (24.4/38.0) في عام 2021 (نسبة الاحتمال < 0.001).

**الاستنتاج:**

انخفض استخدام مضادات البكتيريا لدى المرضى الداخليين بشكل ملحوظ خلال فترة الدراسة. ومع ذلك، فإن النسبة المتزايدة من مضادات الجراثيم المستخدمة كملاذ أخير تظل مثيرة للقلق، وكذلك الفجوة الكبيرة بين نسبة مضادات البكتيريا المستخدمة التي تنتمي إلى مجموعة الوصول والهدف العالمي لمنظمة الصحة العالمية الذي لا يقل عن %60.

## 摘要

**目的:**

分析 2013 年至 2021 年中国三级和二级医院住院患者抗菌药物使用的趋势和模式。

**方法:**

该分析涉及中国抗菌药物监测中心覆盖的医院的季度数据。我们获得了关于医院特征(例如省份、去识别化的医院代码、医院级别和住院天数)和抗菌药物特征(例如通用名称、药物分类、剂量、给药途径和使用量)的信息。我们将住院患者抗菌药物的使用量量化为每 100 人天的限定日剂量数。该分析考虑了世界卫生组织 (WHO) 对抗生素的分类(包括可用类、慎用类和备用类)。

**结果:**

2013 年至 2021 年期间,住院患者抗菌药物使用总量每 100 人天的限定日剂量从 48.8 显著下降到 38.0 (*P* < 0.001)。2021 年,各省之间的差异几乎是双倍的:青海住院患者每 100 人天的限定日剂量为 29.1,西藏为 55.3。在整个研究期间,三级和二级医院最常用的抗菌药物是第三代头孢菌素,约占抗菌药物使用总量的三分之一。碳青霉烯类在 2015 年被列入最常用的抗菌药物分类表中。世界卫生组织分类中最常用的抗菌药物属于慎用类:使用率从 2013 年的 61.3% (29.9/48.8) 显著增加到 2021 年的 64.1% (24.4/38.0) (*P* < 0.001)。

**结论:**

在研究期间,住院患者的抗菌药物使用量显著下降。然而,令人担忧的是,作为“最后手段”的抗菌药物的使用比例不断上升,而属于可用类的抗菌药物的使用比例与世卫组织不低于 60% 的全球目标之间存在着巨大差距。

## Резюме

**Цель:**

Проанализировать тенденции и особенности применения антибактериальных препаратов в больницах третичного и вторичного уровней в Китае в период с 2013 по 2021 год.

**Методы:**

Для анализа использовались ежеквартальные данные больниц, охваченных Центром по надзору за антибактериальными препаратами Китая. Были получены сведения о характеристиках больниц (например, провинция, деидентифицированный код больницы, уровень больницы и количество дней пребывания в стационаре) и характеристиках антибактериальных препаратов (например, непатентованное название, классификация препарата, дозировка, способ применения и объем использования). Проведена количественная оценка применения антибактериальных препаратов как число ежедневных определенных доз на 100 пациенто-дней. При анализе учитывалась классификация антибиотиков по группам свободного доступа (Access), доступа под наблюдением (Watch) и резерва (Reserve) Всемирной организации здравоохранения (ВОЗ).

**Результаты:**

В период с 2013 по 2021 год общий показатель применения антибактериальных препаратов в стационарных условиях значительно снизился с 48,8 до 38,0 условной суточной дозы на 100 пациенто-дней (*P* < 0,001). В 2021 году разница между провинциями увеличилась почти в два раза: 29,1 условной суточной дозы препаратов на 100 пациенто-дней в Цинхае по сравнению с 55,3 в Тибете. Наиболее часто используемыми антибактериальными препаратами в больницах третичного и вторичного уровней в течение всего периода исследования были цефалоспорины III поколения, на долю которых приходилась примерно одна треть от общего количества используемых антибактериальных препаратов. Карбапенемы вошли в список наиболее используемых классификаций антибактериальных препаратов в 2015 году. Наиболее часто используемые антибактериальные препараты по классификации ВОЗ относились к группе используемых под наблюдением (Watch): использование значительно увеличилось с 61,3% (29,9/48,8) в 2013 году до 64,1% (24,4/38,0) в 2021 году (*P* < 0,001).

**Вывод:**

Применение антибактериальных препаратов в стационарных условиях значительно снизилось в течение периода проведения исследования. Тем не менее вызывает обеспокоенность рост доли антибактериальных препаратов, предназначенных для использования в крайнем случае, а также большой разрыв между долей используемых антибактериальных препаратов из группы свободного доступа (Access) и глобальным целевым показателем ВОЗ, который составляет не менее 60%.

## Introduction

The use of antibacterial drugs is a key driver of antibiotic resistance.^1^ China is one of the largest producers and consumers of antibacterials worldwide and, in recent decades, its government has unveiled a series of antibacterial stewardship regulations and clinical guidelines for appropriate antibacterial use.^2^ Although these have had a profound effect, inappropriate antibacterial use and antibacterial resistance remain challenges for the country.

The World Health Organization (WHO) regards surveillance of antibacterial use as a cornerstone of antimicrobial stewardship but, until recently, surveillance has often been ignored and underresourced.^3^ In 2005, the former Chinese health ministry established the Center for Antibacterial Surveillance to strengthen the clinical supervision and management of antibacterial drugs. Initially the surveillance network included only 109 hospitals. By 2021, it covered 2694 tertiary hospitals and 4100 secondary hospitals, which accounted for 90% and 40% of all these hospitals, respectively, in mainland China.^4^^–^^6^

Most previous studies of antibacterial use in China reported regional data,^7^^–^^11^ whereas national studies tended to use procurement data,^12^^–^^14^ which may have been subject to bias given the discrepancy between procurement and usage. Since 2016, the Chinese National Health Commission has issued annual reports of antibacterial use based on data from the Center for Antibacterial Surveillance. However, these data were limited by small sample sizes and covered only 191 tertiary general hospitals. Moreover, the indicators used to evaluate antibacterial use were relatively limited: for example, inpatient indicators included only the total volume and frequency of antibacterial use without more detailed information on the pattern of use.

The aims of this study were to produce up-to-date data on the trends and patterns of inpatient antibacterial use in China’s hospitals, to highlight the challenge of inappropriate antibacterial use, and to provide a benchmark for promoting appropriate antibacterial use and curbing antibacterial resistance.

## Methods

Our study involved aggregated, quarterly data on antibacterial drug use by inpatients at hospitals covered by the Center for Antibacterial Surveillance between 1 January 2013 and 31 December 2021. Data in the nationwide surveillance database covered 31 provinces in China (excluding the Hong Kong Special Administrative Region, the Macao Special Administrative Region and Taiwan, China), as detailed in *China’s Health Statistics Yearbook*.^15^ All secondary and tertiary hospitals that reported antibacterial use during the study period were included; the number of hospitals varied from 1630 in 2013 to 3880 in 2021 (details are available in the online repository).^16^ Participating hospitals uploaded quarterly data using a website-based system.

The data covered antibacterial drugs intended for systemic use, as defined by WHO’s Anatomical Therapeutic and Chemical (ATC) classification J01.^17^ Information on each hospital included: (i) the province; (ii) a de-identified hospital code; (iii) the hospital level; and (iv) the number of inpatient days. Information on each antibacterial drug included: (i) the generic name; (ii) the drug classification; (iii) the specified dosage and form of administration; and (iv) the usage volume (i.e. the number of doses administered per quarter). Antibacterial use was expressed as the number of defined daily doses per 100 patient-days.^17^ For antibacterials without an ATC code or whose defined daily dose was not specified in WHO’s ATC classification, the defined daily dose was imputed from the manufacturers’ recommended daily dose, as approved by China’s Food and Drug Administration.^13^ Antibacterials were further disaggregated at the pharmacological (i.e. ATC level 3), chemical (i.e. ATC level 4) and chemical substance levels (i.e. ATC level 5).

### Data analysis

Trends in, and patterns of, antibacterial use were analysed by ATC level-3 subgroup. In addition, total antibacterial use was also analysed by province and hospital level. The proportion of all antibiotics administered through different routes (i.e. parenteral or oral) and the proportion that belonged to subsections of WHO’s Access, Watch, Reserve (AWaRe) classification were also determined.^18^ The AWaRe classification reflects an antibiotic’s strength and its potential impact on antimicrobial resistance. Antibiotics in the Access group are first- or second-line treatments for common infections and should be widely accessible. Antibiotics in the Watch group should be used only for a limited group of well-defined syndromes and should be closely monitored. Antibiotics in the Reserve group are primarily used as a last resort to treat infections caused by multi- or extensively-drug resistant bacteria. A fourth group – antibiotics not recommended by the AWaRe classification – comprised mainly inappropriate antibiotic combinations with a negative impact on antimicrobial resistance and patient safety. We also added the fifth group – not included antibiotics – that comprised antibiotics which were not in WHO’s AWaRe classification but which were used in China (available in the online repository).^16^ In addition, we identified the most frequently used antibacterial pharmacological classifications (i.e. ATC level 4) that together accounted for 90% of annual antibacterial use and the top 10 individual antibacterial drugs (i.e. ATC level 5) used in each year.

To derive a comparable metric of antibacterial use across time for different types of hospital, province and drug classification, we calculated a compound annual growth rate (CAGR) for antibacterial use:



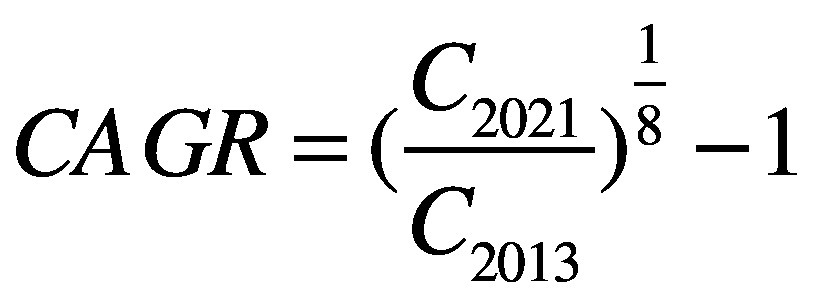

(1)


where *C*_2021_ was the total inpatient antibacterial use in 2021 and *C*_2013_ was the total inpatient antibacterial use in 2013.

The percentage usage of different types and classifications of antibacterials (i.e. ATC level 3 and 4, respectively) and of individual antibacterials (i.e. ATC level 5) in any year was calculated by dividing the usage volume for each type, classification or individual antibacterial, as appropriate, by the total usage volume of all antibacterials in that year.

### Statistical analysis

We report descriptive results. Trends in antibacterial use over time were assessed using linear regression, with antibacterial use as the dependent variable and time as the independent variable.^19^ A *P*-value less than 0.05 was considered statistically significant. Data were managed and analysed in Excel 2019 (Microsoft Corporation, Redmond, United States of America), Stata v. 14.0 (StataCorp LLC, College Station, USA) and OriginPro 2020b (OriginLab Corporation, Northampton, USA).

## Results

### National antibacterial use

National antibacterial use by inpatients in all study hospitals decreased continuously from 48.8 to 38.0 defined daily doses per 100 patient-days between 2013 and 2021 (Table 1 and online repository),^16^ with a CAGR of −3.1% (*P* < 0.001). During the study period, significant decreasing trends were observed in both tertiary (from 47.9 to 37.1 defined daily doses per 100 patient-days; *P*-value: *<* 0.001) and secondary hospitals (from 54.9 to 41.3 defined daily doses per 100 patient-days; *P*-value: *<* 0.001), with the CAGR being larger in secondary than tertiary hospitals: −3.5% versus −3.1%, respectively. Antibacterial use was greater in secondary than tertiary hospitals throughout the study period, except in 2020. Of all antibacterial classifications at ATC level 3, only the J01A classification (tetracyclines) significantly increased in use, from 0.3 to 0.5 defined daily doses per 100 patient-days (CAGR: +5.4%; *P*-value: < 0.001).

**Table 1:** Inpatient antibacterial use, by antibacterial classification and hospital level, China, 2013–2021

Antibacterial classification^a^	Rate of antibacterial use, defined daily doses per 100 patient-days (%)^b^									*CAGR*, %	*P*-value for trend
	Year										
	2013	2014	2015	2016	2017	2018	2019	2020	2021		
**All hospitals**											
All antibacterials	48.8 (100)	47.9 (100)	48.3 (100)	46.8 (100)	44.0 (100)	43.0 (100)	43.0 (100)	39.5 (100)	38.0 (100)	–3.1	< 0.001
J01A: tetracyclines	0.3 (0.7)	0.4 (0.7)	0.4 (0.8)	0.4 (0.9)	0.5 (1.1)	0.5 (1.2)	0.6 (1.3)	0.6 (1.4)	0.5 (1.3)	+5.4	< 0.001
J01B: amphenicols	0.0 (0.0)	0.0 (0.0)	0.0 (0.0)	0.0 (0.0)	0.0 (0.0)	0.0 (0.0)	0.0 (0.0)	0.0 (0.0)	0.0 (0.0)	−20.0	< 0.001
J01C: β-lactam antibacterials	6.8 (13.8)	6.8 (14.2)	6.8 (14.0)	6.6 (14.1)	6.3 (14.3)	6.3 (14.8)	6.6 (15.3)	6.1 (15.3)	6.4 (16.7)	−0.8	< 0.001
J01D: other β-lactam antibacterials	26.3 (53.8)	25.7 (53.7)	25.6 (53.1)	25.4 (54.3)	24.1 (54.7)	23.4 (54.5)	23.2 (54.1)	22.2 (56.2)	21.2 (55.9)	−2.6	< 0.001
J01E: sulfonamides and trimethoprim	0.2 (0.3)	0.2 (0.5)	0.2 (0.4)	0.2 (0.3)	0.1 (0.3)	0.1 (0.2)	0.1 (0.3)	0.2 (0.4)	0.2 (0.4)	−1.0	< 0.001
J01F: macrolides, lincosamides and streptogramins	4.3 (8.9)	4.0 (8.3)	4.2 (8.7)	3.9 (8.3)	3.4 (7.7)	3.0 (7.1)	3.0 (6.9)	1.9 (4.8)	1.9 (4.9)	−10.1	< 0.001
J01G: aminoglycoside antibacterials	1.5 (3.0)	1.3 (2.8)	1.2 (2.5)	1.1 (2.3)	1.0 (2.2)	0.9 (2.2)	0.8 (1.9)	0.8 (1.9)	0.7 (1.8)	−8.8	< 0.001
J01M: quinolone antibacterials	5.9 (12.1)	6.0 (12.6)	6.5 (13.5)	6.0 (12.8)	5.7 (13.0)	5.8 (13.6)	5.9 (13.8)	5.3 (13.4)	4.8 (12.5)	−2.7	< 0.001
J01X: other antibacterials	3.6 (7.3)	3.4 (7.1)	3.3 (6.9)	3.3 (7.0)	2.9 (6.6)	2.8 (6.5)	2.7 (6.4)	2.6 (6.5)	2.4 (6.4)	−4.8	< 0.001
**Tertiary hospitals**											
All antibacterials	47.9 (100)	46.9 (100)	47.6 (100)	46.4 (100)	43.2 (100)	42.7 (100)	42.5 (100)	39.8 (100)	37.1 (100)	–3.1	< 0.001
J01A: tetracyclines	0.3 (0.7)	0.4 (0.8)	0.4 (0.9)	0.5 (1.0)	0.5 (1.2)	0.6 (1.3)	0.6 (1.5)	0.6 (1.6)	0.5 (1.5)	+5.9	< 0.001
J01B: amphenicols	0.0 (0.0)	0.0 (0.0)	0.0 (0.0)	0.0 (0.0)	0.0 (0.0)	0.0 (0.0)	0.0 (0.0)	0.0 (0.0)	0.0 (0.0)	−28.5	< 0.001
J01C: β-lactam antibacterials	6.4 (13.4)	6.5 (13.9)	6.5 (13.6)	6.4 (13.8)	6.0 (14.0)	6.1 (14.4)	6.3 (14.9)	6.0 (15.0)	5.9 (16.0)	−0.9	< 0.001
J01D: other β-lactam antibacterials	25.7 (53.6)	25.1 (53.4)	25.1 (52.7)	25.0 (53.9)	23.5 (54.5)	23.1 (54.0)	22.8 (53.6)	22.2 (55.9)	20.8 (56.0)	−2.6	< 0.001
J01E: sulfonamides and trimethoprim	0.2 (0.4)	0.2 (0.5)	0.2 (0.4)	0.2 (0.4)	0.1 (0.3)	0.1 (0.3)	0.2 (0.4)	0.2 (0.5)	0.2 (0.5)	+0.2	NS
J01F: macrolides, lincosamides and streptogramins	4.3 (8.9)	3.9 (8.3)	4.2 (8.8)	3.9 (8.4)	3.3 (7.6)	3.0 (7.0)	2.9 (6.7)	1.8 (4.6)	1.7 (4.7)	−10.6	< 0.001
J01G: aminoglycoside antibacterials	1.5 (3.1)	1.3 (2.9)	1.2 (2.6)	1.1 (2.4)	1.0 (2.3)	1.0 (2.3)	0.9 (2.1)	0.8 (2.1)	0.7 (2.0)	−8.4	< 0.001
J01M: quinolone antibacterials	5.9 (12.4)	6.1 (12.9)	6.6 (13.9)	6.0 (12.9)	5.7 (13.3)	5.9 (13.8)	6.0 (14.2)	5.4 (13.5)	4.7 (12.6)	−2.9	< 0.001
J01X: other antibacterials	3.6 (7.5)	3.4 (7.3)	3.4 (7.1)	3.3 (7.2)	3.0 (6.9)	2.9 (6.8)	2.9 (6.7)	2.8 (6.9)	2.5 (6.8)	−4.4	< 0.001
**Secondary hospitals**											
All antibacterials	54.9 (100)	53.4 (100)	52.2 (100)	49.1 (100)	48.0 (100)	44.5 (100)	44.9 (100)	38.5 (100)	41.3 (100)	–3.5	< 0.001
J01A: tetracyclines	0.2 (0.4)	0.2 (0.4)	0.2 (0.4)	0.2 (0.3)	0.3 (0.7)	0.3 (0.6)	0.3 (0.7)	0.3 (0.9)	0.3 (0.8)	+4.6	< 0.001
J01B: amphenicols	0.0 (0.0)	0.0 (0.0)	0.0 (0.0)	0.0 (0.0)	0.0 (0.0)	0.0 (0.0)	0.0 (0.0)	0.0 (0.0)	0.0 (0.0)	−0.1	NS
J01C: β-lactam antibacterials	9.0 (16.3)	8.6 (16.1)	8.5 (16.3)	7.8 (15.9)	7.7 (16.0)	7.3 (16.5)	7.6 (17.0)	6.4 (16.7)	8.0 (19.3)	−1.5	< 0.001
J01D: other β-lactam antibacterials	30.1 (54.8)	29.7 (55.6)	28.9 (55.3)	27.8 (56.5)	26.9 (56.1)	25.2 (56.6)	25.2 (56.1)	22.1 (57.4)	23.0 (55.6)	−3.3	< 0.001
J01E: sulfonamides and trimethoprim	0.1 (0.2)	0.2 (0.4)	0.1 (0.1)	0.1 (0.2)	0.1 (0.2)	0.0 (0.1)	0.0 (0.1)	0.0 (0.1)	0.0 (0.1)	−8.3	< 0.05
J01F: macrolides, lincosamides and streptogramins	4.9 (8.8)	4.4 (8.2)	4.4 (8.4)	3.7 (7.5)	3.9 (8.2)	3.2 (7.2)	3.4 (7.5)	2.2 (5.8)	2.3 (5.6)	−8.8	< 0.001
J01G: aminoglycoside antibacterials	1.4 (2.6)	1.3 (2.4)	1.2 (2.2)	1.0 (2.0)	0.8 (1.8)	0.7 (1.6)	0.6 (1.4)	0.5 (1.4)	0.6 (1.4)	−10.4	< 0.001
J01M: quinolone antibacterials	5.8 (10.6)	5.9 (11.0)	6.0 (11.5)	5.9 (11.9)	5.7 (11.8)	5.5 (12.3)	5.6 (12.5)	4.9 (12.7)	5.1 (12.2)	−1.7	< 0.001
J01X: other antibacterials	3.5 (6.3)	3.2 (5.9)	3.0 (5.8)	2.8 (5.7)	2.6 (5.4)	2.3 (5.1)	2.2 (4.8)	2.0 (5.1)	2.0 (4.9)	−6.5	< 0.001

CAGR: compound annual growth rate.

^a^ Antibacterial drugs were classified using the World Health Organization’s Anatomical Therapeutic and Chemical (ATC) classification, level 3.

^b^ Usage of antibacterials in each classification was calculated as a percentage of the total volume of all antibacterial usage in each year.

### Provincial antibacterial use

Antibacterial use was highest in the province of Tibet (55.3 defined daily doses per 100 patient-days), which was 1.9-times greater than in Qinghai, where use was lowest (29.1 defined daily doses per 100 patient-days). There was a downward trend in antibacterial use in 29 of 30 provinces (Tibet was excluded because only 5 years of data were available). However, there was a considerable variation in the trend between provinces: the largest decreasing CAGR was for Shanghai (−5.5%), whereas uniquely there was an increasing CAGR for Hebei (+0.7%). The inpatients antibacterial use in tertiary and secondary hospitals in 31 provinces in 2021 and the corresponding CAGRs between 2013 and 2021 are available in online repository.^16^

### Parenteral and oral antibacterial use

Table 2 reports parenteral and oral antibacterial use by inpatients in tertiary and secondary hospitals and in all hospitals combined between 2013 and 2021. During the study period, the proportion of antibacterials administered parenterally increased significantly from 79.7% to 87.4% (CAGR for the proportion: +1.2%; *P*-value: *<* 0.001), while the proportion administered orally decreased significantly from 20.3% to 12.6% (CAGR: −5.8%; *P*-value: *<* 0.001). The proportion administered parenterally was higher in secondary than tertiary hospitals in every year.

**Table 2:** Inpatient antibacterial use, by administration route and hospital level, China, 2013–2021

Administration route	Proportion of total antibacterial use, %^a^ (defined daily doses per 100 patient-days)									*CAGR*, %	*P*-value for trend
	Year										
	2013	2014	2015	2016	2017	2018	2019	2020	2021		
**All hospitals**											
Oral	20.3 (9.9)	20.1 (9.6)	21.3 (10.3)	19.9 (9.3)	17.5 (7.7)	16.4 (7.1)	15.9 (6.8)	13.2 (5.2)	12.6 (4.8)	−5.8	< 0.001
Parenteral	79.7 (38.9)	79.9 (38.2)	78.7 (38.0)	80.1 (37.5)	82.5 (36.3)	83.6 (36.0)	84.1 (36.1)	86.8 (34.3)	87.4 (33.2)	+1.2	< 0.001
**Tertiary hospitals**											
Oral	20.4 (9.8)	20.3 (9.5)	21.7 (10.3)	20.6 (9.6)	17.6 (7.6)	16.8 (7.2)	16.0 (6.8)	13.0 (5.2)	12.5 (4.6)	−6.0	< 0.001
Parenteral	79.6 (38.1)	79.7 (37.4)	78.3 (37.2)	79.4 (36.9)	82.4 (35.6)	83.2 (35.5)	84.0 (35.7)	87.0 (34.7)	87.5 (32.5)	+1.2	< 0.001
**Secondary hospitals**											
Oral	20.0 (11.0)	18.9 (10.1)	18.9 (9.9)	16.4 (8.1)	17.2 (8.3)	14.8 (6.6)	15.2 (6.8)	13.9 (5.4)	13.0 (5.4)	−5.2	< 0.001
Parenteral	80.0 (43.9)	81.1 (43.3)	81.1 (42.3)	83.6 (41.1)	82.8 (39.7)	85.2 (38.0)	84.8 (38.1)	86.1 (33.1)	87.0 (36.0)	+1.1	< 0.001

CAGR: compound annual growth rate.

^a^ Usage of antibacterials through each administration route was calculated as a percentage of the total volume of all antibacterial usage in each year.

### AWaRe categories

Table 3 reports the use of antibacterials in different AWaRe categories by inpatients in tertiary and secondary hospitals and in all hospitals combined between 2013 and 2021. During the study period, the proportion of antibacterials used that belonged to the Access group fluctuated between 15.1% and 18.4% (*P*-value: 0.099). Overall, most antibacterials used belonged to the Watch group and the proportion that belonged to this group increased significantly from 61.3 in 2013 to 64.1% in 2021 (CAGR: +0.6%; *P*-value: *<* 0.001). In addition, the proportion of antibacterials used that were not recommended increased significantly from 10.6% in 2013 to 11.8% in 2021 (CAGR: +1.3%; *P*-value: *<* 0.05). The proportion of antibacterials used that were not in the AWaRe classification fell from 8.6% in 2013 to 4.9% in 2021 (CAGR: −6.9%; *P*-value: *<* 0.001). The patterns of antibacterial use according to AWaRe classification were similar in secondary and tertiary hospitals, though the proportion of Access group antibacterials used was higher in secondary hospitals: 18.6 to 23.7% compared with 14.3 to 17.4% in tertiary hospitals.

**Table 3:** Inpatient antibacterial use, by AWaRe classification^a^ and hospital level, China, 2013–2021

AWaRe classification^a^	Proportion of total antibacterial use, %^b^ (defined daily doses per 100 patient-days)									*CAGR*, %	*P*-value for trend
	Year										
	2013	2014	2015	2016	2017	2018	2019	2020	2021		
**All hospitals**											
Access	18.4 (9.0)	17.5 (8.4)	16.1 (7.8)	15.7 (7.3)	15.5 (6.8)	15.1 (6.5)	15.2 (6.5)	16.1 (6.4)	17.8 (6.8)	−0.4	NS
Watch	61.3 (29.9)	61.1 (29.2)	62.2 (30.0)	61.9 (29.0)	62.0 (27.3)	62.4 (26.8)	62.8 (27.0)	63.2 (25.0)	64.1 (24.4)	+0.6	< 0.001
Reserve	1.1 (0.5)	0.9 (0.4)	1.0 (0.5)	1.1 (0.5)	1.1 (0.5)	1.3 (0.6)	1.2 (0.5)	1.3 (0.5)	1.3 (0.5)	+2.5	< 0.001
Not recommended	10.6 (5.2)	12.0 (5.7)	12.5 (6.0)	13.7 (6.4)	14.4 (6.3)	14.8 (6.3)	14.9 (6.4)	13.3 (5.2)	11.8 (4.5)	+1.3	< 0.05
Not included^c^	8.6 (4.2)	8.4 (4.0)	8.2 (4.0)	7.6 (3.6)	7.0 (3.1)	6.5 (2.8)	5.9 (2.5)	6.2 (2.4)	4.9 (1.9)	−6.9	< 0.001
**Tertiary hospitals**											
Access	17.4 (8.3)	16.6 (7.8)	15.3 (7.3)	14.9 (6.9)	14.7 (6.3)	14.3 (6.1)	14.4 (6.1)	15.0 (6.0)	16.5 (6.1)	−0.7	< 0.05
Watch	61.7 (29.5)	61.4 (28.8)	62.5 (29.7)	62.2 (28.9)	62.0 (26.8)	62.5 (26.7)	62.9 (26.8)	63.2 (25.2)	64.3 (23.9)	+0.5	< 0.001
Reserve	1.1 (0.5)	1.0 (0.5)	1.1 (0.5)	1.2 (0.5)	1.3 (0.5)	1.5 (0.6)	1.3 (0.6)	1.5 (0.6)	1.6 (0.6)	+4.4	< 0.001
Not recommended	10.9 (5.2)	12.2 (5.7)	12.7 (6.0)	13.8 (6.4)	14.6 (6.3)	14.9 (6.4)	15.1 (6.4)	13.5 (5.4)	12.3 (4.6)	+1.6	< 0.001
Not included^c^	8.9 (4.2)	8.8 (4.1)	8.5 (4.0)	7.9 (3.7)	7.4 (3.2)	6.8 (2.9)	6.3 (2.7)	6.8 (2.7)	5.3 (2.0)	−6.3	< 0.001
**Secondary hospitals**											
Access	23.7 (13.0)	22.2 (11.9)	20.8 (10.9)	19.9 (9.8)	19.1 (9.2)	19.0 (8.5)	18.6 (8.3)	20.3 (7.8)	22.8 (9.4)	−0.5	NS
Watch	58.9 (32.3)	59.5 (31.8)	60.5 (31.6)	60.4 (29.7)	61.6 (29.6)	61.7 (27.5)	62.6 (28.1)	63.2 (24.3)	63.4 (26.2)	+0.9	< 0.001
Reserve	0.8 (0.5)	0.7 (0.4)	0.6 (0.3)	0.7 (0.4)	0.6 (0.3)	0.4 (0.2)	0.4 (0.2)	0.4 (0.1)	0.4 (0.2)	−9.9	< 0.001
Not recommended	9.3 (5.1)	10.8 (5.8)	11.5 (6.0)	12.8 (6.3)	13.3 (6.4)	14.1 (6.3)	14.3 (6.4)	12.3 (4.7)	10.1 (4.2)	+1.0	< 0.05
Not included^c^	7.3 (4.0)	6.8 (3.6)	6.5 (3.4)	6.1 (3.0)	5.3 (2.6)	4.7 (2.1)	4.1 (1.8)	3.9 (1.5)	3.4 (1.4)	−9.1	< 0.001

AWaRe: Access, Watch, Reserve; CAGR: compound annual growth rate; NS: not significant.

^a^ The World Health Organization’s AWaRe classification of antibiotic drugs comprises three groups: Access, Watch and Reserve antibiotics.

^b^ Usage of antibacterials in each AWaRe classification was calculated as a percentage of the total volume of all antibacterial usage in each year.

^c^ A fifth group of not included antibiotics was added to the AWaRe classification, which are ampicillin+probenecid, antofloxacin, apacilin, asmicin, aspoxicillin, cefacidine, cefadroxil/trimethoprim, cefalexin/trimethoprim, cefaloridine, cefathiamidine, cefazolin and sulbactam, cefazuran, cefpimizole, erythromycin cyclic 11,12-carbonate, erythromycin estolate, etimicin, furazolidone, furbucilln, guanacycline, kitasamycin, lenampicillin, meleumycin, morpholinidazole, nemonoxacin, norvancomycin, ornidazole, pipemidic acid, sulbactam, sulfadiazine, sulfadimidine, sulfamethoxazole, ticarcillin clavulanate and tinidazole.

Note: Inconsistencies may arise in some values due to rounding.

### Drugs accounting for 90% of use

The most frequently used antibacterial classifications (ATC level 4), which together accounted for 90% of use in each study year by inpatients in tertiary and secondary hospitals, are shown in Table 4. In 2021: (i) 30.9% of antibacterials used were third-generation cephalosporins (i.e. classification J01DD); (ii) 15.1% were second-generation cephalosporins (J01DC); (iii) 12.5% were fluoroquinolones (J01MA); (iv) 11.6% were penicillins (J01CR); (v) 6.0% were first-generation cephalosporins (J01DB); (vi) 4.2% were imidazole derivatives (J01XD); (vii) 3.8% were macrolides (J01FA); (viii) 3.6% were carbapenems (J01DH); and (ix) 2.9% were penicillins with extended spectrum (J01CA). Between 2015 and 2019, carbapenems (J01DH) replaced other aminoglycosides (J01GB). Neither other aminoglycosides (J01GB) nor carbapenems (J01DH) appeared on the list for secondary hospitals, in contrast to tertiary hospitals.

**Table 4:** Antibacterials accounting for 90% of inpatient use, by hospital level, China, 2013–2021

Antibacterials^a^	Antibacterial use rate, by year																	
	2013		2014		2015		2016		2017		2018		2019		2020		2021	
	Rank	Rate (%)^b,c^	Rank	Rate (%)^b,c^	Rank	Rate (%)^b,c^	Rank	Rate (%)^b,c^	Rank	Rate (%)^b,c^	Rank	Rate (%)^b,c^	Rank	Rate (%)^b,c^	Rank	Rate (%)^b,c^	Rank	Rate (%)^b,c^
**All hospitals**																		
J01DD: third-generation cephalosporins	1	11.4 (23.4)	1	11.3 (23.6)	1	11.9 (24.7)	1	12.1 (25.7)	1	11.8 (26.9)	1	11.8 (27.4)	1	12.0 (28.0)	1	12.1 (30.5)	1	11.7 (30.9)
J01DC: second-generation cephalosporins	2	8.8 (18.0)	2	8.6 (18.0)	2	8.0 (16.5)	2	7.7 (16.5)	2	7.1 (16.2)	2	6.9 (16.1)	2	6.7 (15.7)	2	6.0 (15.2)	2	5.7 (15.1)
J01MA: fluoroquinolones	3	5.9 (12.1)	3	6.0 (12.6)	3	6.5 (13.4)	3	6.0 (12.7)	3	5.7 (13.0)	3	5.8 (13.5)	3	5.9 (13.8)	3	5.3 (13.3)	3	4.7 (12.5)
J01CR: combinations of penicillins, including β-lactamase inhibitors	5	4.4 (9.0)	4	4.6 (9.6)	4	4.6 (9.6)	4	4.7 (10.1)	4	4.5 (10.2)	4	4.6 (10.6)	4	4.8 (11.2)	4	4.2 (10.5)	4	4.4 (11.6)
J01DB: first-generation cephalosporins	4	4.5 (9.2)	5	4.2 (8.7)	5	3.8 (8.0)	5	3.6 (7.7)	5	3.2 (7.3)	5	2.9 (6.8)	5	2.7 (6.3)	5	2.5 (6.3)	5	2.3 (6.0)
J01XD: imidazole derivatives	7	2.9 (6.0)	7	2.8 (5.8)	7	2.7 (5.5)	7	2.5 (5.4)	7	2.2 (5.1)	7	2.1 (4.9)	7	2.0 (4.6)	6	1.8 (4.5)	6	1.6 (4.2)
J01FA: macrolides	6	3.8 (7.8)	6	3.5 (7.4)	6	3.8 (7.9)	6	3.5 (7.5)	6	3.0 (6.9)	6	2.7 (6.3)	6	2.6 (6.1)	7	1.6 (4.0)	7	1.5 (3.8)
J01DH: carbapenems	NA	NA	NA	NA	9	1.2 (2.6)	8	1.4 (3.0)	8	1.4 (3.1)	8	1.4 (3.2)	8	1.4 (3.4)	8	1.5 (3.7)	8	1.4 (3.6)
J01CA: penicillins with extended spectrum	8	1.5 (3.0)	8	1.4 (2.9)	8	1.4 (2.8)	9	1.2 (2.7)	9	1.2 (2.6)	9	1.1 (2.7)	9	1.1 (2.6)	9	1.0 (2.5)	9	1.1 (2.9)
J01GB: other aminoglycosides	9	1.4 (2.9)	9	1.3 (2.7)	NA	NA	NA	NA	NA	NA	NA	NA	NA	NA	NA	NA	NA	NA
**Tertiary hospitals**																		
J01DD: third-generation cephalosporins	1	11.1 (23.1)	1	10.9 (23.2)	1	11.6 (24.3)	1	11.7 (25.2)	1	11.5 (26.5)	1	11.5 (26.9)	1	11.6 (27.4)	1	11.9 (29.8)	1	11.3 (30.3)
J01DC: second-generation cephalosporins	2	8.6 (18.0)	2	8.4 (17.9)	2	7.7 (16.3)	2	7.6 (16.4)	2	6.8 (15.8)	2	6.7 (15.7)	2	6.5 (15.3)	2	6.0 (15.0)	2	5.6 (15.0)
J01MA: fluoroquinolones	3	5.9 (12.3)	3	6.0 (12.9)	3	6.6 (13.8)	3	6.0 (12.9)	3	5.7 (13.2)	3	5.9 (13.8)	3	6.0 (14.2)	3	5.4 (13.5)	3	4.7 (12.5)
J01CR: combinations of penicillins, including β-lactamase inhibitors	4	4.3 (9.0)	4	4.5 (9.7)	4	4.6 (9.6)	4	4.7 (10.1)	4	4.4 (10.2)	4	4.5 (10.6)	4	4.7 (11.1)	4	4.1 (10.3)	4	4.2 (11.4)
J01DB: first-generation cephalosporins	5	4.3 (9.0)	5	4.0 (8.6)	6	3.8 (7.9)	6	3.5 (7.6)	5	3.2 (7.4)	5	2.9 (6.7)	5	2.7 (6.3)	5	2.5 (6.3)	5	2.2 (5.9)
J01DH: carbapenems	NA	NA	NA	NA	8	1.4 (2.9)	8	1.6 (3.4)	8	1.5 (3.6)	8	1.6 (3.6)	8	1.7 (3.9)	7	1.7 (4.3)	6	1.6 (4.3)
J01XD: imidazole derivatives	7	2.9 (6.1)	7	2.8 (5.9)	7	2.6 (5.6)	7	2.5 (5.5)	7	2.2 (5.1)	7	2.1 (4.9)	7	2.0 (4.7)	6	1.8 (4.5)	7	1.6 (4.3)
J01FA: macrolides	6	3.8 (7.9)	6	3.5 (7.5)	5	3.8 (8.0)	5	3.6 (7.7)	6	2.9 (6.8)	6	2.7 (6.3)	6	2.5 (6.0)	8	1.5 (3.8)	8	1.4 (3.7)
J01CA: penicillins with extended spectrum	9	1.3 (2.7)	9	1.2 (2.6)	9	1.2 (2.6)	9	1.2 (2.5)	9	1.0 (2.4)	9	1.1 (2.5)	9	1.0 (2.4)	9	0.9 (2.2)	9	0.9 (2.5)
J01GB: other aminoglycosides	8	1.4 (3.0)	8	1.3 (2.8)	NA	NA	NA	NA	NA	NA	NA	NA	NA	NA	10	0.8 (2.1)	10	0.7 (1.9)
**Secondary hospitals**																		
J01DD: third-generation cephalosporins	1	13.7 (24.9)	1	13.9 (26.0)	1	14.2 (27.1)	1	14.0 (28.6)	1	13.9 (29.0)	1	13.4 (30.0)	1	13.8 (30.7)	1	12.7 (33.1)	1	13.6 (33.1)
J01DC: second-generation cephalosporins	2	9.9 (18.0)	2	9.9 (18.6)	2	9.4 (18.1)	2	8.6 (17.6)	2	8.7 (18.1)	2	8.0 (17.9)	2	7.8 (17.4)	2	6.2 (16.0)	2	6.4 (16.0)
J01MA: fluoroquinolones	3	5.8 (10.5)	3	5.9 (11.0)	3	6.0 (11.5)	3	5.8 (11.9)	3	5.7 (11.8)	3	5.5 (12.3)	3	5.6 (12.4)	3	4.9 (12.7)	3	5.0 (12.7)
J01CR: combinations of penicillins, including β-lactamase inhibitors	5	4.7 (8.6)	4	5.0 (9.4)	4	5.2 (9.9)	4	5.1 (10.3)	4	5.0 (10.4)	4	4.9 (11.0)	4	5.3 (11.8)	4	4.4 (11.3)	4	5.0 (11.3)
J01DB: first-generation cephalosporins	4	5.6 (10.2)	5	5.0 (9.3)	5	4.4 (8.4)	5	4.1 (8.3)	6	3.4 (7.1)	5	3.1 (7.0)	6	2.9 (6.4)	5	2.5 (6.6)	5	2.5 (6.6)
J01FA: macrolides	6	4.2 (7.7)	6	3.8 (7.0)	6	3.8 (7.3)	6	3.2 (6.6)	5	3.5 (7.2)	6	2.8 (6.3)	5	2.9 (6.5)	6	1.8 (4.7)	6	1.8 (4.7)
J01CA: penicillins with extended spectrum	8	2.7 (5.0)	8	2.2 (4.2)	8	2.2 (4.2)	8	1.8 (3.6)	8	1.8 (3.7)	8	1.6 (3.6)	8	1.6 (3.5)	8	1.4 (3.6)	7	1.7 (3.6)
J01XD: imidazole derivatives	7	3.2 (5.8)	7	2.9 (5.5)	7	2.8 (5.3)	7	2.6 (5.3)	7	2.3 (4.9)	7	2.0 (4.5)	7	1.9 (4.2)	7	1.7 (4.5)	8	1.7 (4.5)

NA: not applicable.

^a^ Antibacterial drugs were classified using the World Health Organization’s Anatomical Therapeutic and Chemical (ATC) classification, level 4.

^b^ The rate of antibacterial use in patients is expressed in defined daily doses per 100 patient-days.

^c^ Usage of antibacterials in each classification was calculated as a percentage of the total volume of all antibacterial usage in each year.

### Top 10 antibacterials

**Fig. 1:**
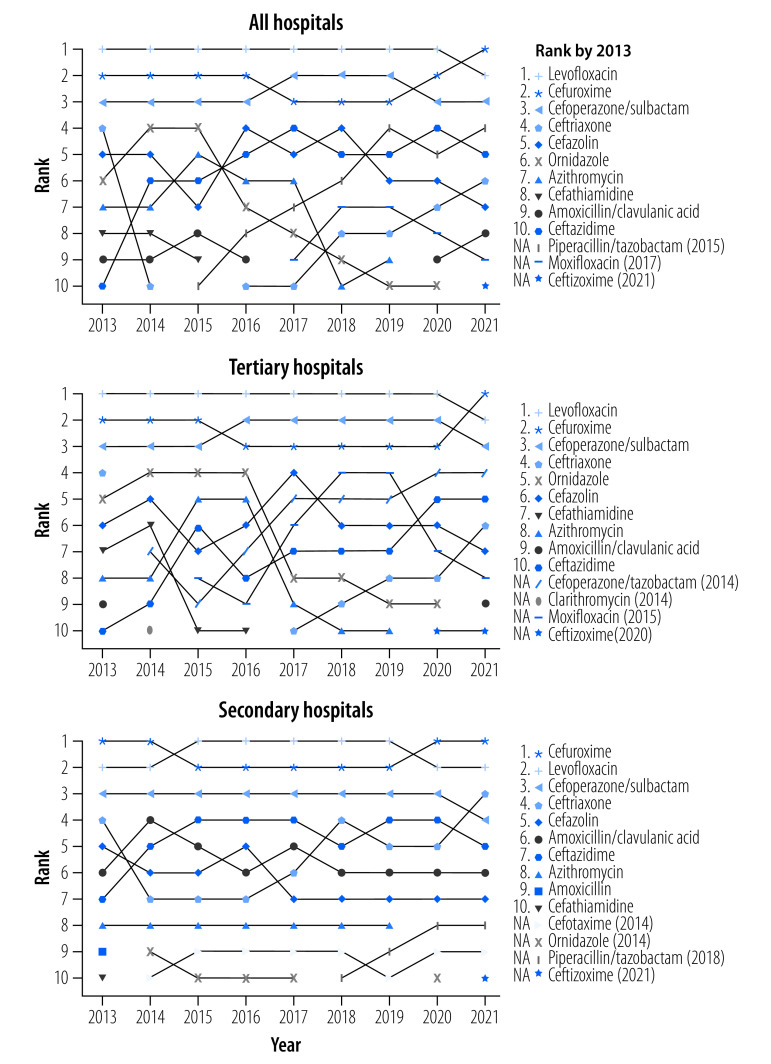
Change in 10 most-used antibacterials among inpatients over time, by hospital level, China, 2013–2021

Table 5 (available at: https://www.who.int/publications/journals/bulletin/) lists the top 10 antibacterials in each year between 2013 and 2021 at tertiary and secondary hospitals and overall, and Fig. 1 shows how the ranking of these antibacterials changed over that time. In 2021, cefuroxime accounted for 9.6% of antibacterials used: corresponding figures for other drugs were 8.9% for levofloxacin, 8.1% for cefoperazone/sulbactam, 6.2% for piperacillin/tazobactam, 5.6% for ceftazidime, 5.3% for ceftriaxone, 4.7% for cefazolin, 3.7% for amoxicillin/clavulanic acid, 3.3% for moxifloxacin and 2.6% for ceftizoxime. Although cefoperazone/sulbactam was not recommended by the AWaRe classification, it has been one of the top three antibacterial treatments in China since 2013. Between 2013 and 2020, levofloxacin was the most frequently used antibiotic but was surpassed by cefuroxime in 2021. The composition of the top 10 antibacterials was similar in tertiary and secondary hospitals.

**Table 5:** Ten most-used antibacterials among inpatients, by hospital level and year, China, 2013–2021

Antibacterial	AWaRe classification^a^	Antibacterial use rate, by year																	
		2013		2014		2015		2016		2017		2018		2019		2020		2021	
		Rank	Rate (%)^b,c^	Rank	Rate (%)^b,c^	Rank	Rate (%)^b,c^	Rank	Rate (%)^b,c^	Rank	Rate (%)^b,c^	Rank	Rate (%)^b,c^	Rank	Rate (%)^b,c^	Rank	Rate (%)^b,c^	Rank	Rate (%)^b,c^
**All hospitals**																			
Cefuroxime	Watch	2	3.9 (8.0)	2	3.8 (7.9)	2	3.3 (6.9)	2	3.1 (6.7)	3	3.1 (7.0)	3	3.1 (7.1)	3	3.0 (7.1)	2	3.3 (8.4)	1	3.6 (9.6)
Levofloxacin	Watch	1	4.6 (9.4)	1	4.6 (9.6)	1	4.9 (10.1)	1	4.5 (9.5)	1	4.1 (9.4)	1	4.1 (9.6)	1	4.1 (9.5)	1	3.7 (9.3)	2	3.4 (8.9)
Cefoperazone/sulbactam	Not recommended	3	2.7 (5.5)	3	3.0 (6.2)	3	2.9 (6.0)	3	3.1 (6.6)	2	3.2 (7.4)	2	3.3 (7.6)	2	3.4 (7.8)	3	3.3 (8.3)	3	3.1 (8.1)
Piperacillin/tazobactam	Watch	NA	NA	NA	NA	10	1.3 (2.7)	8	1.4 (3.0)	7	1.4 (3.3)	6	1.5 (3.6)	4	1.8 (4.2)	5	2.1 (5.2)	4	2.3 (6.2)
Ceftazidime	Watch	10	1.5 (3.0)	6	1.5 (3.1)	6	1.6 (3.4)	5	1.6 (3.4)	4	1.6 (3.7)	5	1.6 (3.8)	5	1.8 (4.1)	4	2.1 (5.3)	5	2.1 (5.6)
Ceftriaxone	Watch	4	2.0 (4.1)	10	1.4 (2.9)	NA	NA	10	1.2 (2.6)	10	1.3 (3.0)	8	1.5 (3.5)	8	1.7 (3.8)	7	1.8 (4.7)	6	2.0 (5.3)
Cefazolin	Access	5	1.7 (3.6)	5	1.6 (3.4)	7	1.6 (3.2)	4	1.6 (3.5)	5	1.6 (3.6)	4	1.7 (3.9)	6	1.7 (4.0)	6	1.9 (4.7)	7	1.8 (4.7)
Amoxicillin/clavulanic acid	Access	9	1.5 (3.1)	9	1.4 (3.0)	8	1.4 (2.8)	9	1.3 (2.8)	NA	NA	NA	NA	NA	NA	9	1.2 (2.9)	8	1.4 (3.7)
Moxifloxacin	Watch	NA	NA	NA	NA	NA	NA	NA	NA	9	1.4 (3.1)	7	1.5 (3.5)	7	1.7 (4.0)	8	1.5 (3.7)	9	1.2 (3.3)
Ceftizoxime	Watch	NA	NA	NA	NA	NA	NA	NA	NA	NA	NA	NA	NA	NA	NA	NA	NA	10	1.0 (2.6)
Cefathiamidine	Not included	8	1.6 (3.3)	8	1.5 (3.0)	9	1.3 (2.7)	NA	NA	NA	NA	NA	NA	NA	NA	NA	NA	NA	NA
Azithromycin	Watch	7	1.7 (3.4)	7	1.5 (3.1)	5	1.6 (3.4)	6	1.6 (3.4)	6	1.4 (3.3)	10	1.3 (2.9)	9	1.3 (3.0)	NA	NA	NA	NA
Ornidazole	Not included	6	1.7 (3.4)	4	1.7 (3.6)	4	1.7 (3.5)	7	1.6 (3.4)	8	1.4 (3.3)	9	1.4 (3.2)	10	1.2 (2.9)	10	1.1 (2.8)	NA	NA
**Tertiary hospitals**																			
Cefuroxime	Watch	2	3.7 (7.7)	2	3.5 (7.5)	2	3.0 (6.4)	3	2.9 (6.2)	3	2.7 (6.4)	3	2.8 (6.5)	3	2.7 (6.4)	3	3.1 (7.8)	1	3.4 (9.1)
Levofloxacin	Watch	1	4.5 (9.4)	1	4.5 (9.5)	1	4.8 (10.0)	1	4.4 (9.4)	1	4.0 (9.3)	1	4.0 (9.4)	1	4.0 (9.3)	1	3.6 (9.0)	2	3.2 (8.6)
Cefoperazone/sulbactam	Not recommended	3	2.6 (5.4)	3	2.8 (6.0)	3	2.7 (5.7)	2	2.9 (6.3)	2	3.1 (7.1)	2	3.1 (7.2)	2	3.2 (7.6)	2	3.3 (8.2)	3	3.1 (8.3)
Cefoperazone/tazobactam	Not recommended	NA	NA	7	1.4 (3.1)	9	1.4 (2.9)	7	1.5 (3.2)	5	1.5 (3.5)	5	1.6 (3.8)	5	1.9 (4.5)	4	2.2 (5.5)	4	2.4 (6.6)
Ceftazidime	Watch	10	1.4 (2.9)	9	1.3 (2.8)	6	1.5 (3.1)	8	1.5 (3.2)	7	1.5 (3.4)	7	1.5 (3.6)	7	1.6 (3.9)	5	2.0 (5.0)	5	2.0 (5.3)
Ceftriaxone	Watch	4	1.8 (3.9)	NA	NA	NA	NA	NA	NA	10	1.2 (2.7)	9	1.3 (3.1)	8	1.5 (3.5)	8	1.6 (4.1)	6	1.7 (4.7)
Cefazolin	Access	6	1.6 (3.4)	5	1.5 (3.3)	7	1.5 (3.1)	6	1.5 (3.3)	4	1.5 (3.6)	6	1.6 (3.7)	6	1.6 (3.9)	6	1.8 (4.6)	7	1.7 (4.6)
Moxifloxacin	Watch	NA	NA	NA	NA	8	1.4 (2.9)	9	1.4 (2.9)	6	1.5 (3.5)	4	1.7 (4.0)	4	1.9 (4.5)	7	1.7 (4.2)	8	1.3 (3.6)
Amoxicillin/clavulanic acid	Access	9	1.4 (2.9)	NA	NA	NA	NA	NA	NA	NA	NA	NA	NA	NA	NA	NA	NA	9	1.1 (3.0)
Ceftizoxime	Watch	NA	NA	NA	NA	NA	NA	NA	NA	NA	NA	NA	NA	NA	NA	10	0.9 (2.4)	10	1.0 (2.7)
Ornidazole	Not included	5	1.7 (3.6)	4	1.7 (3.7)	4	1.7 (3.6)	4	1.6 (3.5)	8	1.5 (3.4)	8	1.4 (3.4)	9	1.3 (3.1)	9	1.2 (2.9)	NA	NA
Azithromycin	Watch	8	1.6 (3.3)	8	1.4 (3.0)	5	1.6 (3.4)	5	1.6 (3.4)	9	1.4 (3.2)	10	1.2 (2.9)	10	1.2 (2.8)	NA	NA	NA	NA
Cefathiamidine	Not included	7	1.6 (3.3)	6	1.5 (3.2)	10	1.3 (2.8)	10	1.2 (2.6)	NA	NA	NA	NA	NA	NA	NA	NA	NA	NA
Clarithromycin	Watch	NA	NA	10	1.3 (2.8)	NA	NA	NA	NA	NA	NA	NA	NA	NA	NA	NA	NA	NA	NA
**Secondary hospitals**																			
Cefuroxime	Watch	1	5.5 (9.9)	1	5.4 (10.1)	2	4.9 (9.4)	2	4.4 (9.0)	2	4.7 (9.8)	2	4.4 (9.9)	2	4.4 (9.7)	1	4.0 (10.4)	1	4.7 (11.3)
Levofloxacin	Watch	2	5.0 (9.1)	2	5.3 (9.9)	1	5.3 (10.2)	1	5.0 (10.2)	1	4.7 (9.8)	1	4.6 (10.3)	1	4.5 (10.0)	2	4.0 (10.4)	2	4.0 (9.7)
Ceftriaxone	Watch	4	3.1 (5.6)	7	2.3 (4.3)	7	2.0 (3.8)	7	2.0 (4.1)	6	2.0 (4.2)	4	2.3 (5.1)	5	2.4 (5.3)	5	2.5 (6.6)	3	3.1 (7.4)
Cefoperazone/sulbactam	Not recommended	3	3.1 (5.7)	3	3.7 (6.9)	3	3.8 (7.3)	3	4.1 (8.3)	3	4.3 (8.9)	3	4.2 (9.3)	3	3.9 (8.8)	3	3.3 (8.6)	4	3.0 (7.3)
Ceftazidime	Watch	7	2.2 (4.0)	5	2.4 (4.5)	4	2.5 (4.8)	4	2.4 (4.9)	4	2.4 (5.0)	5	2.2 (5.0)	4	2.4 (5.4)	4	2.5 (6.6)	5	2.8 (6.8)
Amoxicillin/clavulanic acid	Access	6	2.3 (4.2)	4	2.4 (4.5)	5	2.4 (4.7)	6	2.3 (4.7)	5	2.2 (4.6)	6	2.1 (4.7)	6	2.2 (4.8)	6	2.1 (5.3)	6	2.6 (6.2)
Cefazolin	Access	5	2.5 (4.6)	6	2.3 (4.4)	6	2.2 (4.1)	5	2.3 (4.8)	7	2.0 (4.1)	7	2.0 (4.5)	7	2.0 (4.6)	7	2.1 (5.3)	7	2.0 (4.9)
Piperacillin/tazobactam	Watch	NA	NA	NA	NA	NA	NA	NA	NA	NA	NA	10	1.1 (2.5)	9	1.5 (3.3)	8	1.6 (4.2)	8	1.9 (4.7)
Cefotaxime	Watch	NA	NA	10	1.5 (2.8)	9	1.5 (2.8)	9	1.4 (2.8)	9	1.3 (2.7)	9	1.2 (2.8)	10	1.3 (2.9)	9	1.2 (3.1)	9	1.5 (3.7)
Ceftizoxime	Watch	NA	NA	NA	NA	NA	NA	NA	NA	NA	NA	NA	NA	NA	NA	NA	NA	10	1.0 (2.4)
Azithromycin	Watch	8	2.0 (3.7)	8	1.9 (3.6)	8	1.8 (3.4)	8	1.7 (3.4)	8	1.9 (3.9)	8	1.5 (3.3)	8	1.6 (3.5)	NA	NA	NA	NA
Ornidazole	Not included	NA	NA	9	1.5 (2.8)	10	1.5 (2.8)	10	1.4 (2.8)	10	1.2 (2.5)	NA	NA	NA	NA	10	0.9 (2.3)	NA	NA
Amoxicillin	Access	9	1.8 (3.2)	NA	NA	NA	NA	NA	NA	NA	NA	NA	NA	NA	NA	NA	NA	NA	NA
Cefathiamidine	Not included	10	1.6 (2.8)	NA	NA	NA	NA	NA	NA	NA	NA	NA	NA	NA	NA	NA	NA	NA	NA

AWaRe: Access, Watch, Reserve; NA: not applicable.

^a^ The World Health Organization’s AWaRe classification of antibiotic drugs comprises three groups: Access, Watch and Reserve antibiotics. A fifth group of not included antibiotics was added to the classification.

^b^ The rate of antibacterial use in patients is expressed in defined daily doses per 100 patient-days.

^c^ Usage of each individual antibacterial was calculated as a percentage of the total volume of all antibacterial usage in each year.

NA: not applicable

Note: Antibacterial drugs were classified using the World Health Organization’s Anatomical Therapeutic and Chemical (ATC) classification, level 5. Further information on these antibacterials’ use rate by hospital level and year can be found in Table 5.

### COVID-19 period

During the coronavirus disease 2019 (COVID-19) pandemic, antibacterial use overall fell by 8.1% from 43.0 defined daily doses per 100 patient-days before the pandemic in 2019 to 39.5 defined daily doses per 100 patient-days in 2020, and continued to decrease to 38.0 defined daily doses per 100 patient-days in 2021. However, the pattern was different in secondary hospitals: total antibacterial use decreased sharply from 44.9 defined daily doses per 100 patient-days in 2019 to 38.5 defined daily doses per 100 patient-days in 2020 and then increased to 41.3 defined daily doses per 100 patient-days in 2021 (Table 1 and the online repository).^16^ Although the overall use of parenteral antibacterials decreased to 33.2 defined daily doses per 100 patient-days in 2021, the proportion of antibacterial use that was parenteral increased to 87.4% (Table 2). The proportion of antibacterials that belonged to the Access group started to increase in 2020, to 16.1%, and rose to 17.8% in 2021, whereas the proportion of drugs that were not recommended decreased from 13.3% in 2020 to 11.8% in 2021. The pattern was the same in secondary and tertiary hospitals. There was no change in the antibacterial classifications that comprised 90% of total antibacterial use during the COVID-19 period. However, ceftizoxime and amoxicillin/clavulanic acid first entered the top 10 in tertiary hospitals in 2020 and 2021, respectively (Table 5).

## Discussion

During the study period, inpatient antibacterial use decreased significantly in tertiary and secondary hospitals in China and was lower than has been reported in both low- and middle-income countries, such as Ethiopia (81.6 defined daily doses per 100 patient-days),^20^ Eritrea (79.5 defined daily doses per 100 patient-days) and Nepal (113.7 defined daily doses per 100 patient-days),^21^^,^^22^ and high-income countries, such as Australia (93.6 defined daily doses per 100 patient-days),^23^ Israel (84.0 defined daily doses per 100 patient-days),^24^ the Netherlands (85.7 defined daily doses per 100 patient-days),^25^ Saudi Arabia (94.5 defined daily doses per 100 patient-days) and Sweden (65.7 defined daily doses per 100 patient-days).^26^^,^^27^ This finding suggests that the antibacterial stewardship system probably reduced antibacterial overuse in China.^28^ In 2018, the Chinese government stated that antibacterial stewardship should gradually move from being administratively led to a comprehensive, multidisciplinary collaboration and that it should focus on high-risk groups, such as paediatric patients, pregnant women and elderly people.^29^ Our study found that interprovincial variability in antibacterial use was substantial, which means that surveillance at the provincial level is important for identifying the determinants of antibacterial use, for assessing the prevalence of antibacterial-resistant pathogens and for guiding appropriate interventions. Moreover, the potential impact of interprovincial variability should be tackled more comprehensively under the OneHealth approach to ensure the risk of antibacterial resistance is minimized.^30^

Our study found that the proportion of antibacterials used by inpatients that belonged to the Access group in the AWaRe classification was under 20%. Although the Chinese government has promoted the appropriate use of narrow-spectrum antibacterials and has strived to increase their proportional use to international levels,^29^ the proportion is still far below the target set by WHO (i.e. Access group antibacterials should account for no less than 60% of total antibacterial use) and is lower than in most countries that reported this figure.^18^^,^^31^ In China, antibacterial formulary restriction lists were established at the provincial level in 2012.^5^ Antibacterials were divided into three groups according to the prevalence of resistance, safety, drug efficacy and cost: (i) non-restricted; (ii) restricted; and (iii) highly-restricted.^5^ The Chinese classification categorized many drugs in the AWaRe classification’s Watch group (e.g. levofloxacin) as non-restricted, which may have contributed to the greater proportionate use of broad-spectrum antibacterials we found. We also found that ornidazole and cefathiamidine, which were not included in the AWaRe classification, were two of the top 10 antibacterials used in China. This finding highlights the importance of ensuring that the categorization of antibacterials is evidence-based and that antibacterials are used appropriately.

Although inpatient antibacterial use decreased more in secondary than tertiary hospitals, use per patient–day was consistently higher in secondary hospitals. This finding is concerning given that secondary hospitals had fewer inpatients with less severe disease than tertiary hospitals, according to *China’s Health Statistics Yearbook*.^15^ Moreover, the proportion of antibacterials administered parenterally was much higher than the proportion administered orally in both secondary and tertiary hospitals and was increasing. The proportion was higher than that found in previous studies based on procurement data, which included outpatients and primary care.^13^^,^^32^ The higher proportion of parenteral administration among inpatients may have been due to the hierarchical nature of the health-care delivery system:^33^ patients with mild or moderate disease may have been encouraged to seek treatment in primary care, with more severe patients attending higher-level hospitals.^34^ In addition, the prohibition of drug infusion for outpatients (except for children's hospitals) was gradually introduced from 2016 onwards,^35^ which may have further increased parenteral antibacterial use.

Cephalosporins, especially second- and third-generation cephalosporins, were consistently the most-used antibacterials in China. In Europe, New Zealand, Switzerland and the United Kingdom, in contrast, penicillins have been the most-used antibacterials.^19^^,^^31^^,^^36^^–^^38^ The difference between China and other countries might be explained by the setting: in China, most antibacterials are given to hospital inpatients, whereas in other countries most are given to ambulatory patients and to those treated in general practice or in the community. Moreover, cephalosporins are recommended by Chinese national guidelines for most perioperative prophylaxis indications, which may have increased their use.^39^ We found that levofloxacin was the most-used antibacterial between 2013 and 2020. Ease of administration may have contributed to levofloxacin’s use. Penicillins, in contrast, require time-consuming skin allergy testing before administration. Carbapenems (ATC classification: J01DH) became one of the most frequently used antibacterial drug classes between 2015 and 2021 despite being classified as highly restricted and requiring preauthorization before use. The National Health Commission now requires hospitals to establish specialized management for carbapenem.^40^ The increase in carbapenem use we found (from 2.6% of all antibacterials in 2015 to 3.6% in 2021) may have been due to a rise in the prevalence of infections with extended-spectrum, β-lactamase-producing, gram-negative bacteria. For example, carbapenem use was observed to increase in parallel with the increased incidence of carbapenem-resistant *Klebsiella pneumoniae* infection seen in tertiary hospitals.^33^ Hence, it is important to have dynamic data on temporal trends and patterns in antibacterial use and antibacterial resistance to identify correlations between them.

Our finding that inpatient antibacterial use decreased between 2013 and 2021 contrasts with our previous, population-weighted, antibiotic use research,^13^ which demonstrated a 38.2% increase in antibiotic consumption in hospitals between 2011 and 2018. There are several possible reasons for this discrepancy. First, the hospitals sampled in this and previous studies were not identical, which may have introduced sample bias. Second, the procurement data used in previous studies may not have reflected actual drug utilization. Third, the metric adopted in previous studies was the number of daily defined doses per 1000 inhabitant–days, which was calculated by dividing the number of daily defined antibiotic doses procured by the population size. A manufacturing statistics yearbook – the *Report of pharmaceutical market development in China (2018)* – showed that the pharmaceutical market for anti-infection drugs has grown continuously since 2011.^41^ In parallel, the number of inpatients in Chinese tertiary hospitals almost tripled between 2010 and 2017, which implies that demand for antibiotics increased. Meanwhile, according to *China’s Health Statistics Yearbook*,^15^ the population grew only slightly from 1.35 billion in 2011 to 1.40 billion in 2018. As a result, antibiotic use per 1000 inhabitants would be expected to increase because antibiotic use (the numerator) grew much faster than the population (the denominator).

The sudden drop in inpatient antibacterial use we found in 2020 was probably due to China’s COVID-19 prevention and control strategies. The successive series of isolation and protection measures introduced would have diminished the number of hospitalizations and, consequently, inpatient antibacterial use.^42^ Researchers showed that, after adjusting for provincial COVID-19 cases, antibiotic use remained constant. The impact of the COVID-19 pandemic on population-level antibiotic consumption in China could mainly be attributed to the decline in medical services caused by epidemic prevention and control measures, therefore, rather than to the treatment of COVID-19 cases.^43^ The rebound of antibacterial use in secondary hospitals in 2021, then, may reflect the adaptation of the medical system to these measures. However, more detailed studies are needed to improve understanding of the impact of the pandemic at the population level.

Our study has several limitations. First, the lack of data on antibacterial use before stewardship initiatives were introduced makes it difficult to be sure the initiatives were responsible for the trends over time. Second, the representativeness of the hospitals included in the study may have been affected by selection bias because participation in the surveillance network was voluntary. However, according to *China’s Health Statistics Yearbook*,^15^ tertiary and secondary hospitals in the study accounted for over 63.5% and 16.5%, respectively, of the total number of these hospitals in mainland China. Third, the Center for Antibacterial Surveillance’s database does not cover antibacterial use in primary health care or in the community, hence we could not assess overall antibacterial use. Our analysis focused on hospital inpatient antibacterial use so we did not have to consider import or manufacturing data, which use different measures. However, our results were robust within the scope of the study. Fourth, only aggregated data were available for analysis. Nonetheless, the Center for Antibacterial Surveillance has an internal quality control process and the National Health Commission has conducted training programmes to improve data collection, which should ensure data quality. Fifth, the use of aggregated data meant that no patient-level information on the clinical use of antibacterials was available and, therefore, we could not assess the appropriateness of antibacterial use in individuals.

In conclusion, our study found that antibacterial use by inpatients at tertiary and secondary hospitals in China decreased significantly between 2013 and 2021, probably reflecting the positive effects of introducing antibacterial stewardship. However, the rising proportion of last-resort antibacterials used is concerning, as is the large gap between the proportion of antibacterials used in China that belonged to the Access group and WHO’s global target of no less than 60%. More effort is needed to ensure that surveillance data provide the impetus to optimize antibacterial use and curb antibacterial resistance, especially in the post-pandemic era.
